# Apexification in Non-Vital Teeth with Immature Roots: Report of Two Cases

**Published:** 2014-12-24

**Authors:** Rogério Vieira Silva, Frank Ferreira Silveira, Eduardo Nunes

**Affiliations:** a *Department of Dentistry, Pontificial Catholic University of Minas Gerais, Belo Horizonte, Minas Gerais, Brazil*

**Keywords:** Apexification, Apical plug, Calcium Hydroxide, Mineral Trioxide Aggregate, MTA

## Abstract

Apexification is a method of inducing apical closure for non-vital immature permanent teeth. During this treatment a mineralized barrier is induced [with long term calcium hydroxide (CH) treatment]; or artificially created [with mineral trioxide aggregate (MTA) plug]. This article describes two cases of apexification in immature necrotic teeth treated with these two different techniques. After 6 years of follow-up, clinical and radiographic control showed that both treatments were successful.

## Introduction

Bacteria can harbor within the complexities of root canal system and dentinal tubules, as a result of caries and coronal-root fractures. These microorganisms and their byproducts are responsible for initiating/maintaining a periapical inflammatory process [1].

Endodontic treatment of immature teeth with incompletely formed roots can result in complications that necessitate special precaution. The apical anatomy in these teeth is characterized by greater width at the apical portion compared to the cervical and the absence of apical constriction that challenge the clinician with difficulty in determining and staying within the working length (WL) [2, 3]; also the very thin root dentin walls render the tooth susceptible to fracture. In such cases, it is necessary to induce the closure of the apical foramen with mineralized tissue or to create an artificial apical barrier to allow for condensation of the root filling material and promote an apical seal [4].

Apexification is a method of inducing apical closure in non-vital teeth with incomplete root formation and works by forming a mineralized barrier. Induction of this calcified barrier is usually performed using calcium hydroxide (CH) mixed with a vehicle [1]. Depending on the stage of root development, complete formation of the calcified apical barrier may be prolonged, and multiple care sessions (from 6 to 24 months) are needed to achieve this goal [4]. After formation of the calcified apical barrier the root canal is obturated with conventional techniques.

An alternative treatment is the use of an artificial barrier with mineral trioxide aggregate (MTA) apical plug [2, 3]. MTA is a biomaterial with excellent biocompatibility and good sealing property that is useable even in the presence of moisture. Moreover, both the patient and the practitioner benefit from the use of MTA as the total treatment time is reduced [5].

This article describes two cases of apexification in immature non-vital teeth managed with both of the aforementioned treatments.

## Case Report

***Induction of calcified apical barrier***

**Figure 1:**
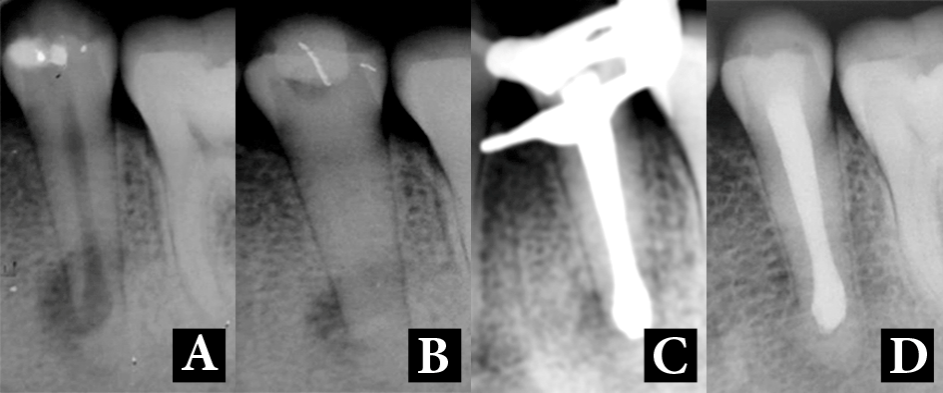
*A*) Preoperative radiography of the mandibular left second premolar; *B*) Root canal dressing with calcium hydroxide paste; *C*) Canal obturation after induction of apical barrier; *D*) Six-year follow-up

A 15-year-old female came to the endodontic clinic at Pontifical Catholic University, Belo Horizonte, MG, Brazil. Clinical examination revealed the presence of a draining sinus tract in the periradicular area of the mandibular left second premolar. Radiographic examination revealed the presence of a periapical lesion located lateral to the apex of the tooth #35 and incomplete root development (Figure 1A). Pulp vitality tests, including the cold test (Endo-Ice, The Hygenic Corp., Akron, OH, USA) and an electric pulp testing (Analytic Technology, Redmond, WA, USA) elicited a negative response, which was suggestive of pulp necrosis and chronic apical periodontitis. Considering the width of the apical foramen the treatment plan included induction of a calcified apical barrier with CH (apexification). After informing the patient and her parents, an informed consent was obtained from them. 

During the first session, the coronal access was prepared with a #1557 drill (KG Sorensen, Barueri, SP, Brazil) and complete isolation was immediately achieved. The canal was then located and the WL was determined with an electronic apex locator (Endex, Osada Electric Co., Tokyo, Japan) and confirmed with radiographic images. Then the canal was prepared with crown-down instrumentation complemented with 2.5% NaOCl (Lenza Farmacêutica Ltda, Belo Horizonte, MG, Brazil) irrigation which was carried with a 25-gauge needle connected to a disposable 5-mL syringe with simultaneous aspiration to avoid accidental injection of NaOCl into the periodontal tissue. The canal patency was as large as a #80 hand K-file.

Then the canal was filled with creamy mixture of CH powder (pro analysis, Labsynth Produtos para Laboratório LTDA, Diadema, SP, Brazil) and saline and the tooth was temporarily restored (Figure 1B). One month later, after ensuring the disappearance of the sinus tract, the intracanal medicament was removed with hand filing and 2.5% NaOCl irrigation activated with ultrasonic tip (ENAC, ST 21, ENAC OE-W10, Osada Co., Tokyo, Japan). Then the canal was filled with CH paste with a thick consistency and the patient was dismissed; four months later in the third treatment session, the paste was refreshed.

At the fourth session which was 9 months later, the root canal was irrigated with 3 mL of 17% EDTA (Lenza Farmacêutica Ltda, Belo Horizonte, MG, Brazil) for 1 min. Final irrigation was done with 2.5% NaOCl, and the canal was dried. Formation of the a mineralized barrier in the apical region was carefully and gently verified with a #60 K-file (Dentsply Maillefer, Ballaigues, Switzerland). Obturation of the root canal was carried out using a #80 gutta-percha cone (Odus de Deus, Belo Horizonte, Brazil) and PCS EWT sealer (Pulp Canal Sealer with Extended Working Time, Kerr Sybron Dental Specialties, Califórnia, USA). Then the thermoplasticized vertical condensation of gutta-percha was employed using the medium tip of a System B electric device (SybronEndo, CA, USA) supplemented with the use of an Obtura II device (Corp, Fenton, MO, USA) (Figure 1C). 

At the 6-year follow-up, the patient was clinically asymptomatic and new bone formation could be observed in the periapical region. There were also radiographic signs of lateral and apical root closure (Figure 1D).

***MTA Apical plug***

A 15-year-old male attended the endodontic clinic at Pontifical Catholic University, Belo Horizonte, MG, Brazil, complaining of the fractured maxillary left central incisor after a traumatic event the time of which he could not recall. Attempts to bond the fractured fragment and restoration with resin could be observed on clinical examination. Radiographic examination revealed a immature apex and a radiolucent area surrounded the periapical region of tooth #21. The tooth was not responsive to cold and electrical pulp testing which confirmed pulp necrosis and chronic apical periodontitis. The treatment plan included single visit apexification with MTA apical plug. An informed consent was taken from the patient and his parents.

**Figure 2:**
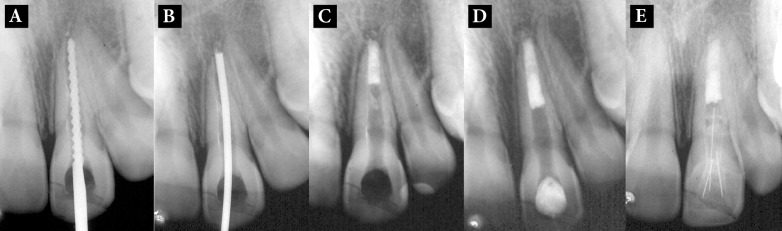
*A*) Operative radiography of the maxillary left central incisor with a #140 K-file; *B)* MTA-specific points inside the canal; *C) *placement of the MTA apical plug; *D)* Canal backfilling *E)* Six years later

Conventional coronal access was prepared with a #1557 drill and absolute isolation was obtained with rubber dam and dental floss. The canal was then located and the WL was determined with an electronic apex locator (J. Morita corp., Tokyo, Japan) and was confirmed with a radiographic image and a #140 K-file (Figure 2A). Then the canal was gently irrigated with 2.5% NaOCl carried with a 25-gauge needle connected to a disposable syringe with simultaneous aspiration. In the same session, the canal was dried and filled with creamy CH paste carried into the canal using a Lentulo spiral (Dentsply Maillefer, Ballaigues, Switzerland). The tooth was temporarily restored and three weeks later, under complete isolation, the intracanal medicament was removed with hand files and NaOCl irrigation which was aspirated with a capillary tip (Ultradent do Brasil, Indaiatuba, SP, Brazil). The canal was dried with absorbent paper points. Tooth-colored MTA (Ângelus, Londrina, PR, Brazil) was mixed with provided liquid according to the manufacturer's instructions. A radiographic image was taken with the MTA-specific points (Ápice, Belo Horizonte, Brazil) placed in the canal to ensure correct positioning (Figure 2B). Using Schilder pluggers (Odous de Deus, Belo Horizonte, MG, Brazil) a 4 mm-thick MTA apical plug was gently applied and packed into the apical segment and confirmation radiography was taken (Figure 2C). The plug was rechecked with a surgical microscope (DF Vasconcelos, SP, Brazil). To provide the hydration required for primary setting of MTA, a moist #60 paper cone was placed in contact with MTA for 15 min. Then, the additional 3 mm of the canal was obturated using an Obtura II system and PCS EWT sealer (Figure 2D). 

Six-year radiographic control showed that the treatment was successful and new bone formation could be observed in the periapical region which is a sign of optimal seal (Figure 2E).

## Discussion

Historically, induction of a calcified apical barrier with long-term intracanal CH medication, used to be the most common technique for inducing biological sealing in teeth with incompletely formed apices [6, 7]. Despite its proven clinical success, this technique has some disadvantages such as the prolonged time period for completion of the treatment, which requires the patient's cooperation, especially regarding the need for several follow-up appointments [5]. Moreover, there is a risk of recontamination of the root canal system through the temporary sealing material [8] and cervical tooth fracture during or after treatment is likely [1].

MTA was introduced in 1993 at the Loma Linda University (USA) initially as a root-end filling material [1]. MTA is an excellent alternative for the conventional CH apexification due to its favorable physicochemical properties and biocompatibility [5, 9], especially because it enables treatment to be completed more quickly, with high clinical success [10, 11].

However, apexification with MTA apical plug requires specific facilities such as points and carriers to facilitate its insertion, and correct adaptation within the ideal apical limit may be more difficult in extremely large foramina. Nevertheless, studies have reported that the MTA placement technique using passive ultrasonic vibration can improve the marginal adaptation of the material [12]. Moreover, the surgical microscope enables increased lighting and makes viewing and insertion of the MTA easier, although radiographic confirmation at this stage cannot be overlooked [1]. 

One should take into consideration that the application of a dressing with CH paste for the treatment of teeth with an incomplete apices will facilitate decontamination of the pulp cavity. Moreover, the application of MTA in teeth with divergent apices becomes difficult or even infeasible due to the difficulty in adapting this material to this segment. In these situations, the prior use of dressings with CH becomes necessary to create conditions that facilitate MTA setting and improves its properties [13, 14].

As described in the reported cases, the use of these two biomaterials (CH and MTA) in teeth with incomplete root formation demonstrated clinical and radiographic success at follow-up with restoring the masticatory function and dental aesthetics.

## Conclusion

It can be concluded that both techniques have favorable outcomes by restoring the tooth function and esthetic. But considering the less treatment sessions and less chair time of MTA apical plug this treatment can be the choice for immature necrotic teeth.
